# Postoperative Computed Tomographic Assessment of the Complete Resection of an Infiltrative Lipoma Compressing the Spinal Cord in a Dog

**DOI:** 10.3390/vetsci10100593

**Published:** 2023-09-26

**Authors:** Junyoung Kim, Kitae Kim, Dayoung Oh, Hyunwook Myung, Jihye Choi, Junghee Yoon

**Affiliations:** 1 N Animal Medical Center, Seoul 02732, Republic of Korea; smilevet78@snu.ac.kr; 2 College of Veterinary Medicine and the Research Institute for Veterinary Science, Seoul National University, Seoul 08826, Republic of Korea; imsono@snu.ac.kr; 3 Veterinary Health Center, University of Missouri, Colombia, MO 65211, USA; kkbvm@missouri.edu; 4 Department of Environmental and Radiological Health Sciences, College of Veterinary Medicine and Biomedical Sciences, Colorado State University, Fort Collins, CO 80523, USA; dayoung.oh@colostate.edu; 5 NEL Animal Medical Center, Anyang 14065, Republic of Korea; doctor_myung@daum.net

**Keywords:** postoperative computed tomography, infiltrative lipoma, body fat measurement, dog

## Abstract

**Simple Summary:**

Infiltrative lipomas have a tendency to infiltrate adjacent muscle and fibrous tissue without metastasis, such as muscle; connective tissue; bone; and, in rare cases, peripheral nerves and the spinal cord. Incomplete surgical excision of infiltrative lipoma has been shown to increase recurrence. This case report describes a dog with an extensive infiltrative lipoma impinging on the vertebral canal, compressing the spinal cord and extending into the surrounding muscle layers and thoracic cavity. The mass was surgically removed, and subsequent postoperative computed tomography with the automated fat measurement technique confirmed complete removal. The patient’s prognosis could be assessed through postoperative computed tomography, and there has been no recurrence during the 2-year follow-up period.

**Abstract:**

Infiltrative lipomas, which are locally invasive tumors composed of well-differentiated adipocytes, are histologically identical to lipomas but have a tendency to infiltrate adjacent muscle and fibrous tissue without metastasis, such as muscle; connective tissue; bone; and, in rare cases, peripheral nerves and the spinal cord. They differ from liposarcomas yet also exhibit neoplastic cell infiltration and often recur despite surgical removal. A 10-year-old spayed Maltese female dog presented with hindlimb paresis and back pain for two months. Computed tomography and magnetic resonance imaging revealed an extensive fatty mass impinging on the vertebral canal, compressing the spinal cord, and extending into the surrounding muscle layers and thoracic cavity. The mass was surgically removed, and subsequent postoperative computed tomography confirmed complete removal of the mass using Vitrea^®^ advanced visualization fat measurement. Histopathological analysis confirmed that the mass was an infiltrative lipoma. The patient’s symptoms completely resolved after surgery, with no recurrence reported at the 2-year follow-up. This case highlights the benefits of using postoperative computed tomography combined with the automated fat measurement technique to determine whether reoperation is necessary or to predict patient prognosis by identifying potential residual lipoma post-surgery.

## 1. Introduction

Infiltrative lipomas, which are locally invasive tumors composed of well-differentiated adipocytes, are histologically identical to lipomas but have a tendency to infiltrate adjacent muscle and fibrous tissue without metastasis, such as muscle; connective tissue; bone; and, in rare cases, peripheral nerves and the spinal cord [1,2,3]. It remains unclear why infiltrative lipomas exhibit more invasive behavior than simple lipomas despite being derived from the same adipocytes [3]. They differ from liposarcomas yet also exhibit neoplastic cell infiltration and often recur despite surgical removal [4]. A definitive diagnosis for infiltrative lipoma relies on histological analysis [2]. In dogs, they are typically located in the ventral thoracic wall and extremities but can also originate from the head and neck muscle regions [2,5]. Reports have also demonstrated infiltrative lipomas extending from the paraspinal musculature into the spinal extradural space, causing neurologic deficits [2,5]. As clinical signs are location-specific, complete surgical excision is the best way to treat symptomatic infiltrative lipomas [2,6,7].

Advanced imaging techniques are essential for accurately assessing the distribution, shape, size, and location of the mass before surgical intervention or radiation therapy [3]. Computed tomography (CT) and/or magnetic resonance imaging (MRI) have been employed for preoperative planning in cases of infiltrative lipoma in dogs [5,6]. However, incomplete excision of infiltrative lipoma has been shown to increase recurrence [1,2,3], in both human [8] and veterinary medicine, with a recurrence rate of 36–50% reported previously [1,3,9]. By comparison, after simple lipoma, the local recurrence rate can be as low as 2% [3]. In one study, the duration from surgical resection to recurrence was approximately 6 months, and the extent of tumor infiltration increased with each operation [3]. In addition, the growth rate of infiltrative lipoma is more rapid than that of lipoma [3]. Thus, achieving complete excision of an infiltrative lipoma is paramount to minimizing the risk of recurrence.

The present case report describes the removal of an extensive infiltrative lipoma within the thoracic vertebral canal, whereby complete excision was confirmed using CT immediately after surgery.

## 2. Case Presentation

**Figure 1:**
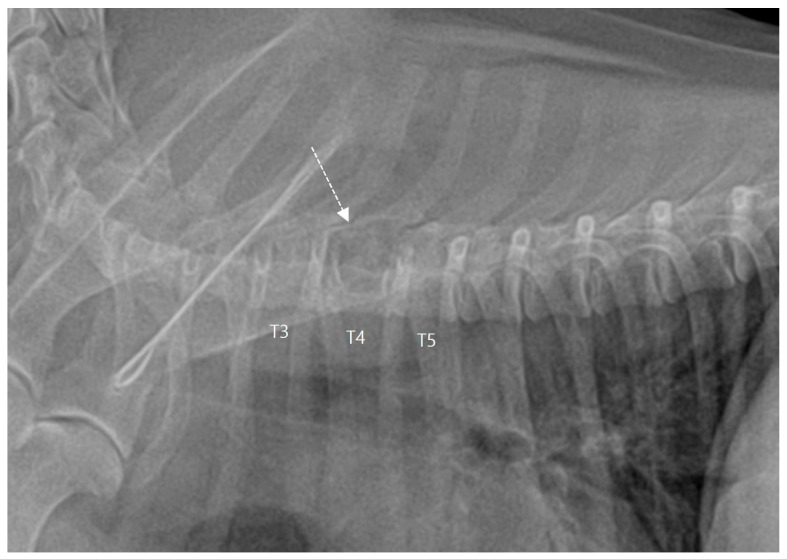
Lateral radiograph of the thoracic spine on the right side revealing a radiolucent lytic lesion of the fourth thoracic vertebra (T4; dotted arrow). T: thoracic vertebra.

**Figure 2:**
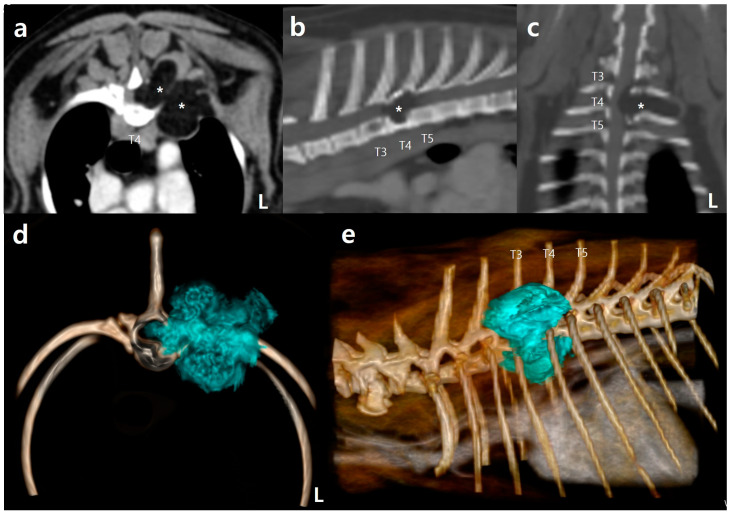
Computed tomography (CT) images indicating the possibility of infiltrative lipoma: (**a**) post-contrast CT image with the transverse view (soft-tissue window) showing a homogeneous, hypoattenuating, well-circumscribed mass (asterisks) infiltrating the surrounding muscle layers and the fourth thoracic vertebra (T4) and extending into the vertebral canal. The mass was not contrast-enhanced; (**b**) post-contrast CT with the sagittal multi-planar reformatting (MPR) view (bone window) demonstrating a hypoattenuating bone lesion (asterisk) in T3, T4, and the craniodorsal end of the T5 vertebral body; (**c**) post-contrast CT with the coronal MPR view (bone window). The hypoattenuating mass (asterisk) compresses the spinal cord, and the cranially protruding mass compresses the vertebral canal, extending into the left lamina of T3; (**d**) three-dimensional (3D) reconstruction image showing a mass (blue embedded) infiltrating from T3 to T5, as viewed from the front of the patient; (**e**) 3D reconstruction image produced by describing the mass (blue embedded), as viewed from the left side of the patient.

A 10-year-old spayed female Maltese dog weighing 2.65 kg presented with a 2-month history of abnormal gait, hind limb paresis, and mild back pain on palpation. The dog was tentatively diagnosed with intervertebral disc disease and was treated with strict cage rest, methylprednisolone sodium succinate, and weekly laser therapy. However, the symptoms worsened, and the dog was referred to the N Animal Medical Center. Neurological examination revealed upper motor neuron signs, rear limb paraparesis, loss of proprioception and motor function, and spinal walking. Complete blood counts and serum biochemistry showed no abnormalities. Thoracic radiography revealed a distinct radiolucent lesion in the fourth thoracic vertebra (T4), raising suspicion of an aggressive lesion, such as a tumor (Figure 1). Pre- and post-contrast whole-body CT scans were performed using a 16-multislice CT scanner (Brivo CT385, GE Hangwei Medical Systems Co., Ltd., Beijing, China) with the following CT parameters: tube voltage, 120 kVp; tube current, 150 mAs; slice thickness, 0.5 mm; rotation, 0.5 s; and helical pitch, 0.813. A contrast medium (300 mg/I/mL; Omnipaque^™^; GE Healthcare, Seoul, Republic of Korea) was administered into the cephalic vein at a rate of 2.0 mL/s via a power injector (OptiVantage^®^ DH, Mallinckrodt, Dublin, Ireland). The CT images revealed a well-defined, homogeneous, hypoattenuating mass with a density of −119 ± 8 Hounsfield units (HU), indicative of lipoma. This fatty mass also exhibited extensive distribution spanning from T3 to T5, measuring approximately 2.3 cm (height) × 1.9 cm (length) × 1.7 cm (width), and in the vertebral canal and surrounding muscle layers on the left side of T4. Moreover, destruction of the left T3–T5 pedicles and T3 and T4 vertebral bodies was observed, with subsequent invasion into the thoracic cavity (Figure 2). Three-dimensional (3D) volume-rendering images were reviewed on a dedicated viewing station using specialized software (Vitrea 7.12, Vital Images, Inc., Minnetonka, MN, USA) and highlighted the extensive distribution of the mass. In addition, utilizing Vitrea^®^ advanced CT fat measurement allowed separate and automatic assessment of visceral and subcutaneous fat tissues. No evidence of metastasis in the whole body, including the lungs, other vertebrae, and thoracic or abdominal lymph nodes, was found using CT.

**Figure 3:**
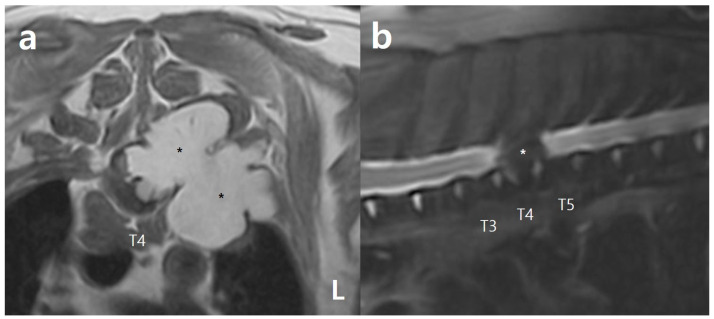
Magnetic resonance imaging (MRI) further supported the possibility of infiltrative lipoma: (**a**) a T1-weighted transverse image of the mass in the T4 region. Note the hyperintense mass (asterisks) significantly compressing the spinal cord and extending into the adjacent muscles; (**b**) a T2-weighted fat-saturated sagittal image showing a hypointense mass (asterisk) in the T4 region, suggesting lipoma. Note the hyperintense change in the spinal cord adjacent to the mass compared to the spinal cord located away from the mass, indicating edema or gliosis secondary to spinal compression.

MRI was performed using a 1.5 T MRI scanner (Toshiba Excelart Vantage Atlas; Toshiba Medical Systems Corp., Tochigi, Japan) with 3 mm slice thickness to obtain detailed information about the spinal cord and soft tissues surrounding the fatty mass in the thoracic vertebrae. T1- and T2-weighted MRI revealed a hyperintense mass that extended into several muscle layers surrounding the left side of T4 (the multifidus, longissimus thoracis, and thoracic iliocostal muscles; Figure 3). The mass further protruded into the vertebral canal and exerted substantial compression on the spinal cord between T3 and T5, predominantly through the left aspect of the destructed vertebrae (Figure 3). In addition, mild T2 hyperintensity was visible in the spinal cord parenchyma surrounding the mass, which was deemed indicative of spinal edema or gliosis arising as a secondary compression-related response (Figure 3). T2-weighted fat-saturated images confirmed the fatty composition of the mass, with the most pronounced compression observed at T4. No significant evidence indicative of myelomalacia, such as prominent spinal cord swelling or an extended spinal cord parenchymal lesion, was identified. The CT and MRI examination findings strongly suggested an infiltrative fatty mass that invaded into the adjacent muscles, connective tissues, and vertebrae, rather than a simple lipoma or liposarcoma, causing spinal cord compression and the patient’s neurological signs.

**Figure 4:**
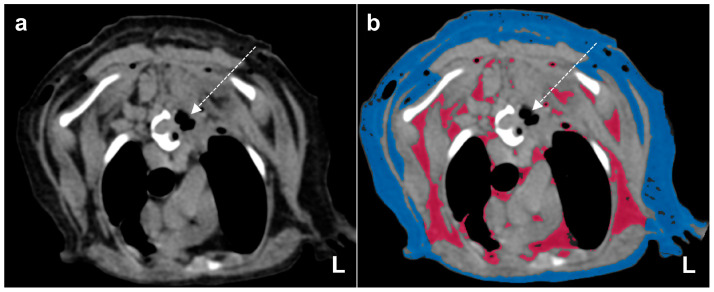
(**a**) Postoperative computed tomography (CT) images at the T4 level (axial view in soft-tissue window); (**b**) the Vitrea^®^ Advanced Visualization CT fat measurement technique was used to visualize the fat tissue, which was colored using an attenuation range of −30 to −150 Hounsfield Units. Red and blue areas indicate visceral fat and subcutaneous fat, respectively. Gas and soft-tissue opacities with no residual fatty mass are present around the surgical site (dotted arrows).

**Figure 5:**
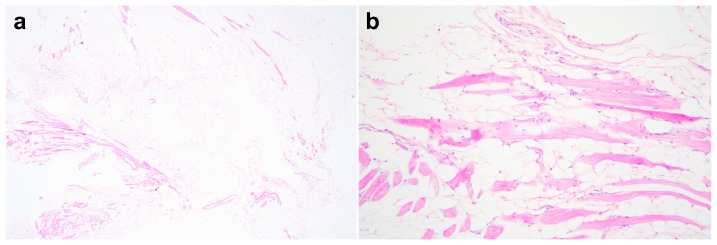
Photomicrographs of the excised tissue specimen ((**a**): 40×, (**b**): 200×) show that most of the cells consisting of the mass are well-differentiated adipocytes. The large, clear vacuoles replace the cytoplasm, and the nuclei are placed eccentrically and are compressed. The proliferative adipocytes are seen infiltrating into the muscle and collagen bundles, and small delicate blood vessels are scattered among the adipocytes. Mitotic figures are not seen in the tissues. The neoplastic adipocytes show no malignant characteristics, such as pleomorphism, anaplasia, dysplasia, or cellular atypia. These histopathological findings indicate an infiltrative lipoma.

Surgical excision of the fatty mass was performed to relieve spinal cord compression. A left hemilaminectomy at T4 and thoracotomy were performed through the ventrolateral approach, due to suspected invasion of the fatty mass into the thoracic wall and vertebral body. The borders of the mass were carefully evaluated, and the mass, which extended into T3, could not be fully visualized, due to limited visibility of the cranially projecting mass situated on the left side of T3 within the vertebral canal. Furthermore, unlike general fatty masses, this fatty mass lacked a well-defined capsule. Therefore, the regions with a clear margination between the mass and the surrounding soft tissues were dissected and excised using a blunt separation technique. Further, in the regions where the boundaries were indistinct or the visibility of the mass was limited, debulking using an ultrasonic surgical instrument was performed. This approach was chosen to remove the residual masses as much as possible, reducing the risk of causing further damage to the spinal cord and vertebral body. Despite our best efforts, we had doubts about the complete surgical removal of the residual lipoma. Therefore, immediately following the surgery, postoperative CT scans were obtained to verify the complete excision of the mass and to assess the necessity for any further surgical intervention. Residual fatty mass verification was manually conducted at a range of −30 to −150 HU and was further evaluated using a repeated automated body fat measurement technique using specialized software (Vitrea 7.12, Vital Images, Inc., Minnetonka, MN, USA) (Figure 4). On postoperative CT images, we only observed gas resulting from the surgery and soft-tissue opacities measuring in the range of 30 to 50 HU at the surgical site (Figure 4). Subsequent intraoperative assessment confirmed the absence of any remaining fatty mass. The patient’s recovery from anesthesia proceeded uneventfully. Histopathological analysis revealed that the mass was an infiltrative lipoma that had invaded the adjacent muscle layers from T3 to T5 (Figure 5). Following surgery, the dog exhibited voluntary weight-bearing movements consistently throughout the day. Subsequent neurological assessment demonstrated normal postural responses and pain reactions, with the exception of a mild reduction in proprioceptive positioning of the pelvic limbs. By postoperative day 10, the dog had regained complete neurological function. At the time of writing this report 2 years after discharge, no recurrence of neurological signs has been reported.

## 3. Discussion

This report describes a case of an infiltrative lipoma with extensive neurological symptoms involving the vertebral column and surrounding muscle tissues and spinal cord compression and demonstrates the clinical usefulness of postoperative CT with the automated body fat measurement technique (Vitrea^®^ Advanced CT fat measurement) for confirming the complete removal of adipose tissue immediately following surgical excision.

On the CT images, the fatty mass in this case exhibited a uniform hypoattenuating fat density without contrast enhancement and had infiltrated the surrounding muscle layers extensively, leading to a strong suspicion of an infiltrative lipoma rather than a lymphosarcoma or a simple lipoma. In this case, the infiltrative lipoma led to thoracic vertebra destruction and spinal cord compression. In the literature, seven cases of infiltrative lipoma invading the vertebral canal and causing neurological deficits have been documented [2,3,5,6,7,10]. Among them, four cases successfully regained neurological function post-surgery [2,5,6,7]. Another study described a dog with progressive neurological symptoms, which resulted in death seven months after a second mass resection surgery [3]. In the two additional reports, the owners of the dogs opted for euthanasia [10]. In the present case, the neurological deficits were fully reversed following surgical intervention. Both CT and MRI data were utilized to plan the surgical procedure. This approach ensured a comprehensive understanding of the mass’s size, extent, and location and facilitated the evaluation of any secondary spinal cord injuries. Moreover, a subsequent postoperative CT scan in conjunction with automated body fat measurement during post-processing provided confirmation of complete resection of the extensively distributed mass with a high level of confidence, which might not have been clearly identifiable during surgery.

Although MRI is more sensitive than CT for detecting microscopic fat, the scanning time is significantly longer; hence, postoperative CT was performed to avoid extending the anesthetic duration. Nonetheless, CT imaging with automated body fat measurement helps distinguish fat masses with greater sensitivity compared to standard CT methods, highlighting the clinical utility of this technique and its potential as a viable alternative to MRI.

While the implementation of intraoperative CT scanning has been reported in human medicine [11], research in veterinary medicine has been limited. A human case report suggested that in patients with juvenile nasopharyngeal angiofibroma, contrast-enhanced CT scanning 2.8 ± 1.8 days after surgery could detect residual tumor cells even when the resection was deemed complete [12]. However, in dogs, anesthesia is required to control respiration and obtain image optimization for thorough residual mass evaluation. Since older patients experience increased anesthesia-related mortality and tumor development vulnerability, the CT procedure described in this report may help prevent poor outcomes.

Nevertheless, this study has some limitations, which include the potential for recurrence in the future, and follow-up CT scans after discharge have not been conducted thus far. Despite the patient undergoing complete lipoma resection and remaining free of recurrence for 2 years, whether vertebral body lysis was a direct consequence of bone infiltration or secondary to pressure-related mechanisms, such as vascular supply compromise, remains unclear, limiting prognostic accuracy [1]. Histopathological analysis is considered the gold standard for determining the adequacy of excision [13]. Nevertheless, postoperative CT provides valuable insights and assists in complete removal, especially for tumors with infiltrative characteristics or for those at difficult locations that may not be well visualized or evaluated during surgery. While postoperative CT cannot guarantee prevention, it can help to mitigate the risk of recurrence. Furthermore, additional scanning offers surgeons more information about the presence of any residual mass, enabling them to plan further excision before the patient’s anesthetic recovery. In this case, we did not conduct postoperative contrast-enhanced CT since the mass was presumed to be an infiltrative lipoma based on preoperative CT and MRI findings; however, contrast-enhanced CT can yield more detailed information based on the specific tumor type. In our approach, we used an automated body fat measurement technique with CT that has been widely applied as an advanced imaging tool in both human and veterinary medicine for estimating abdominal fat accumulation and assessing obesity [14]. By leveraging the mass’s lipid composition, we employed this technique to postoperatively evaluate and confirm the absence of any residual infiltrative lipoma after manual examination.

## 4. Conclusions

The use of postoperative CT with automatic fat measurement facilitated the complete removal of an infiltrative lipoma extending into multiple muscle layers and the vertebral canal in a dog. This procedure is particularly advantageous over conventional CT during the removal of masses with extensive infiltration or those located in challenging areas that cannot be visualized during surgery. It can also help predict patient prognosis by identifying potential postoperative residual lipomas and determining whether reoperation is necessary. While the approach of combining automatic fat measurement was highly beneficial in this case, it is imperative to assess and determine its clinical utility by applying it to a larger cohort of veterinary patients.

## Data Availability

The data that support the findings of this study are available from the corresponding author upon reasonable request.
